# SnakeStrike: A Low-Cost Open-Source High-Speed Multi-Camera Motion Capture System

**DOI:** 10.3389/fnbeh.2020.00116

**Published:** 2020-08-03

**Authors:** Grady W. Jensen, Patrick van der Smagt, Egon Heiss, Hans Straka, Tobias Kohl

**Affiliations:** ^1^Graduate School of Systemic Neurosciences (GSN-LMU), Ludwig-Maximilians-University Munich, Munich, Germany; ^2^argmax.ai, Volkswagen Group Machine Learning Research Lab, Munich, Germany; ^3^Department of Artificial Intelligence, Faculty of Informatics, Eötvös Lórand University, Budapest, Hungary; ^4^Institute of Zoology and Evolutionary Research, Friedrich-Schiller-University of Jena, Jena, Germany; ^5^Department Biology II, Ludwig-Maximilians-University Munich, Munich, Germany; ^6^Chair of Zoology, Technical University of Munich, Freising, Germany

**Keywords:** motion capture, high-speed, opensource, tracking, snake, strike

## Abstract

Current neuroethological experiments require sophisticated technologies to precisely quantify the behavior of animals. In many studies, solutions for video recording and subsequent tracking of animal behavior form a major bottleneck. Three-dimensional (3D) tracking systems have been available for a few years but are usually very expensive and rarely include very high-speed cameras; access to these systems for research is limited. Additionally, establishing custom-built software is often time consuming – especially for researchers without high-performance programming and computer vision expertise. Here, we present an open-source software framework that allows researchers to utilize low-cost high-speed cameras in their research for a fraction of the cost of commercial systems. This software handles the recording of synchronized high-speed video from multiple cameras, the offline 3D reconstruction of that video, and a viewer for the triangulated data, all functions previously also available as separate applications. It supports researchers with a performance-optimized suite of functions that encompass the entirety of data collection and decreases processing time for high-speed 3D position tracking on a variety of animals, including snakes. Motion capture in snakes can be particularly demanding since a strike can be as short as 50 ms, literally twice as fast as the blink of an eye. This is too fast for faithful recording by most commercial tracking systems and therefore represents a challenging test to our software for quantification of animal behavior. Therefore, we conducted a case study investigating snake strike speed to showcase the use and integration of the software in an existing experimental setup.

## Introduction

High-speed video recording is a common tool to visualize and subsequently quantify fast behavioral performances such as in snakes (Kardong and Bels, 1998; Young, 2010; Herrel et al., 2011; Penning et al., 2016; Ryerson and Tan, 2017), other fast moving animals (Patek et al., 2004; Tobalske et al., 2007; Seid et al., 2008), or insect flight (e.g. Altshuler et al., 2005; Boeddeker et al., 2010; Geurten et al., 2010; Straw et al., 2011). However, in most snake studies only one camera or a maximum of two are used to capture such rapid motion, with the one exception of a recent study where multiple cameras with only moderate temporal resolution, were used to investigate locomotor maneuvers (Gart et al., 2019). Software such as DLTdv (Hedrick, 2008), Tracker (Open Source Physics)^1^, ImageJ (Rasband, 1997-2018), or Didge (Alistair Cullum, Creighton University) have usually been used to process the captured images. These open source solutions are suitable tools to use when capturing with a single camera and with a known distance to the recorded object(s), with an exception for DLTdv as it performs triangulation when combined with calibration information provided by a different software. Single camera capture, however, creates some limitations. Using a mirror allows a single camera to perceive multiple views of the snake such as done by Kardong and Bels (1998), but any time a single camera is used to capture three-dimensional (3D) information, the camera must be placed in a setup that is stereotypically well-defined in a way that the distances such as between camera sensors or from the camera sensor to the object are known. The inflexibility of these well-defined setups can be troublesome for the use in multiple experiments, requires extra expertise, and entails extra costs for building and storage.

Motion capture technology using multiple infrared cameras has been available for experimental studies already for decades. While one of the principal fields of employment for these systems was and still is the capture of human motion, this technology has been used in more recent years for the tracking of animal locomotion (Dahmen and Zeil, 1984; Fry et al., 2000; Straw et al., 2011; Tian et al., 2011; Theunissen and Dürr, 2013; Robie et al., 2017; Theunissen et al., 2017). Systems such as Vicon, Optitrack, Motion Analysis, Qualisys, or XSense are largely comparable and use infrared reflective spherical surface markers on the subject of interest that are tracked by multiple spatially fixed cameras and allow triangulating the positions of various body parts in virtual 3D space. In contrast, active marker-based tracking systems such as Dari Motion, Myomotion, NDI, or marker-less systems, commonly use depth information and a wire-frame, or similar, model of the tracked object mostly for applications involving humans. These model-based systems are expensive with costs that range from $10,000 to $100,000, though some open source algorithms are available for human pose estimation in video recordings (e.g. OpenPose, Cao et al., 2018, DeepPose, Toshev and Szegedy, 2014, ArtTrack, Insafutdinov et al., 2017, and DeeperCut, Insafutdinov et al., 2016). While commercial systems work well with low reconstruction error and ease of use, this technology is rather insufficient for high-speed motion capture, mostly because of the typically low maximal camera frame rates of 100–250 Hz. Accordingly, details of ultrafast movements such as strikes of rattlesnakes, which from initiation to target contact are completed within ∼0.05 s (Kardong and Bels, 1998; Penning et al., 2016) require a camera with a capture frame rate well beyond 200 Hz. At such a frame rate, and using a state-of-the-art tracking procedure, optical recordings of a rattlesnake strike would comprise a mere 10 frames of triangulated trajectory. Accordingly, many details about the kinematic profile would be unavailable and thus invisible apart from the fact that raw images are usually not stored. Though often done to save disk space as well as to minimize bandwidth saturation, it prevents any re-analysis of the triangulated motion trajectory.

Here, we present a multi-camera system that allows high-speed motion capture of ultrafast animal movements such as snake strikes using low-priced cameras with high frame rates of 750 Hz and sufficient spatial resolution. The developed software provides a suite of functions that encompass the entirety of data collection, processing, and storage of motion capture with a special focus on processing speeds for high-speed camera capture. Another advantage of using a single software for the data recording and processing pipeline is that the amount of ambiguity and pitfalls that inexperienced motion capture practitioners could encounter is decreased. The amount of data that is generated by high-speed cameras grows quite rapidly with the number of cameras, their speed, and their resolution. Handling this large amount of data often causes problems and can be vastly time consuming when large data sets are processed without performance optimization. This software processes the data to the full extent of the available computer system resources; a feature not available in other tracking software such as DLTdv Hedrick (2008), but which greatly diminishes the time required for processing. Although this system was developed in order to capture snake strike motion dynamics, it can easily be employed for motion studies of other animals than snakes. SnakeStrike is an open source framework written to allow users to harness the power from other open source libraries for image manipulation, camera interaction, and computer vision (Bradski, 2000; Schroeder et al., 2006; Guennebaud and Jacob, 2010; Rusu and Cousins, 2011; Moulon et al., 2013). Thus, besides assisting in the resource-intense and time-critical initial collection of images at high frame rates with multiple cameras, SnakeStrike performs subsequent offline image processing for triangulation and data visualization. Color thresholding is used for marker identification, allowing simultaneous tracking of multiple animals or body parts when markers with different colors are used. If infrared cameras are used, then infrared markers can subsequently also be used. Marker types need not be spherical or 3D in form. Something as simple as a piece of colored tape can be used, and no wire-frame, or similar model, is required. A major advantage of SnakeStrike is the storage of all original images as reference. This permits repeated off-line data re-interpretation in case new automatic tracking methods or sequential modeling methods become available. Because of the open source code and the modular structure of SnakeStrike, other annotation and pose estimation tools such as DeepLabCut (Mathis et al., 2018) or LEAP (Pereira et al., 2019) can be incorporated into the processing pipeline.

## Materials and Methods

In this section we describe the requirements to use the system and how we fulfilled those requirements, how to use the software from a user’s perspective, as well as provide a high-level overview of how the images are processed such that 3D triangulated points of the markers become available. The order of the sections follows the order the user will generally interact with the interface. This order is reinforced by the software to create a consistent pipeline for the user. Where appropriate we include suggestions for solutions to problems that can arise during use. Furthermore, we describe the experimental setup used for each of our two experimental settings.

### Hardware Requirements

For effective recording of high-speed videos for 3D tracking, it is important to use cameras that are fast enough to capture every detail of the motion of interest and can function together. The only absolute requirements to run the SnakeStrike software are a 64-bit computer running 64-bit Linux. To ensure faithful data transfer to the computer, enough bandwidth on a single bus or multiple busses for the communication protocol is required to save data without dropping individual frames. Therefore, it should be thoroughly calculated which camera communication protocol (e.g. USB3, Ethernet, etc.) is most beneficial given the respective requirements. The only requirements are that the camera adheres to the GenICam standard and has an application programming interface (API). Since very large amounts of data need to be transferred and stored, a computer equipped with sufficient sized RAM and hard drives that have enough storage space to store the recorded raw images is required. However, the precise camera and computer configuration generally depends on the speed of the motion of interest and total recording time necessary to capture every detail. An example data set of only 1 second of recording time from a setup equipped with 5 USB 3.0 cameras with a resolution of 640 × 480 pixels and using lossless data compression requires ∼700 MB of disk space, while ∼3.2 GB of RAM is the minimal requirement for the images from the data capture, assuming that the images are returned from the camera in RGB8 format. If the format is changed to something like Bayer BG8, then the amount of required RAM decreases by a third. Sufficient RAM for running the software should also be included in the calculation. Since AC powered lights flicker when recorded with high-speed cameras, lights for the experimental setup need to have a flicker frequency that is higher than the camera speed or are non-flickering. Note that not all LED lights are non-flickering.

To satisfy secondary requirements of our experiments, we used a computer with multiple CPU cores, 64 GB RAM, and several Terabytes of available hard disk storage. Furthermore, we used multiple USB 3.0 cameras. Consequently, a PCI card that expanded the available USB 3.0 ports and ensured that each new port had its own controller was utilized. A separate controller for each port guaranteed that the port would not share bandwidth with other USB 3.0 ports. When running a high-speed camera, it is very easy to saturate these buses with a single camera, let alone multiple. For triggering the cameras, a software trigger is usually available, although this does not guarantee synchronized images when using a USB connection. If a hardware trigger is required while using USB cameras, an external hardware trigger must be added.

We chose Basler Ace acA1300-200uc cameras (Basler AG, Ahrensburg, Germany), which have a maximum image size of 1280 × 1024 pixels. At full spatial resolution, the maximum speed is 203 Hz, however, if the resolution is decreased to 640 × 480 pixels, a frame rate of 750 Hz can be achieved. With camera speeds this high, light flickering of the illumination can be a major issue. Accordingly, surgical lights were used when recording snake strikes in the 3D *X*-ray setup. However, AQ Aquaflora 54-watt fluorescent bulbs (D-D The Aquarium Solution Ltd., Ilford, United Kingdom) are cost-efficient and have successfully been applied in initial tests. When available, non-flickering LED-technology can be used as an alternative. The used cameras were connected to the computer by USB 3.0. To exclude potentially dropped images due to saturation of the bus, a Startech PEXUSB3S44V card (StarTech.com, London, ON, Canada) was used. For correct triangulation during our experiments, USB camera synchronization was essential. Accordingly, a Labjack U3 (Lakewood, CO, United States) AD/DA converter with custom-built housing to trigger the cameras *via* three available digital ports was used. A maximum of three cameras can be triggered per port, without critical attenuation of the TTL signal. As long as the camera speed is less than 25,000 fps, i.e. one frame per 40 μs, the 20 μs delay between each of the pulses is short enough to ensure a quasi-simultaneous image recording from all triggered cameras. Thus, recorded images are saved as the same frame. If only one trigger is available, another solution to allow all cameras to be triggered is to use a buffer amplifier; this prevents the signal from being affected by load currents and ensures a truly synchronous trigger.

### User Interface

SnakeStrike is currently only available for 64-bit Linux. The C++ source code and installation instructions are available at “https://github.com/gwjensen/SnakeStrike”, while the main user and code documentation can be found at “https://gwjensen.github.io/SnakeStrike/”. Since compiling C++ source code with many dependencies is not an easy task, a docker image with the required dependencies as well as SnakeStrike pre-installed is available on DockerHub at gwjensen/snake_strike with the requisite source for building the Docker image manually located at “https://github.com/gwjensen/SnakeStrikeDocker”. Information regarding plugins or specific functions is available at “https://gwjensen.github.io/SnakeStrike/”.

**FIGURE 1:**
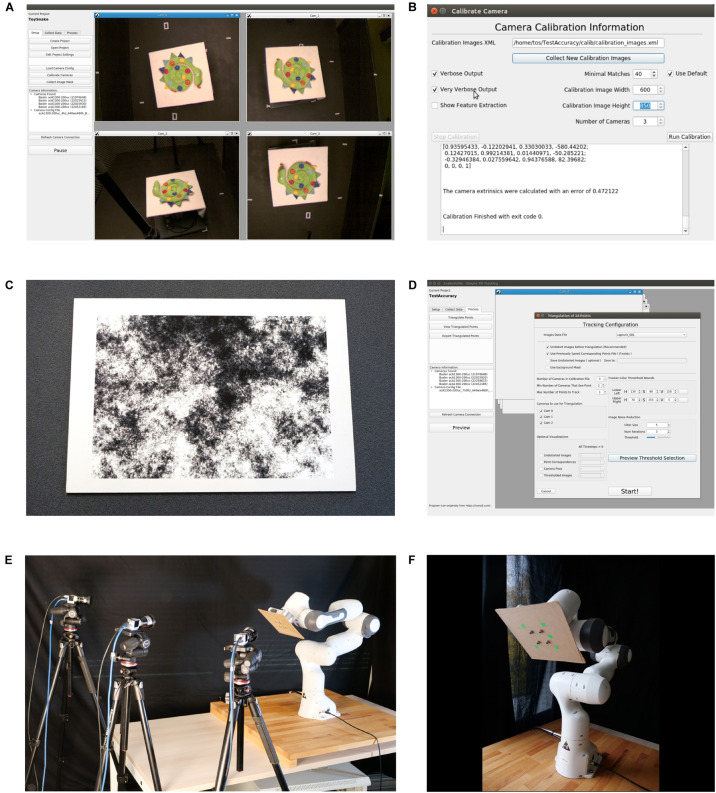
User Interface of the SnakeStrike software and view of the experimental setup used for Study 1. **(A)** Main screen of the software depicting simultaneous previews from four connected cameras. **(B)** Calibration dialog window after a successful calibration attempt. **(C)** DIN A1-sized calibration image adopted from Li et al. (2013), affixed to a 1 cm thick cardboard for calibration. **(D)** Example of the dialog window used to triangulate the markers encountered in the images. **(E)** Overview of the experimental setup used for study 1, depicting the arrangement of three low-cost Basler cameras and a Franka Emika Panda robotic arm. **(F)** Close-up of the robotic arm with green markers affixed to a piece of fiberboard.

The main method of interacting with SnakeStrike is through a basic graphical user interface (GUI). The interface manages folder structure and encapsulates the many steps behind the actions of calibration, capture, and triangulation tasks. The functionality of the interface is divided into three separate tabs in the top left corner of the window (Figure 1A). First, the user creates a new project/experiment, where a project/experiment comprises a single data collection. This ensures consistent data annotation output and reduces workload for the user by automatically organizing created files. After the project is created, new options become available guiding the user along the GUI. For instance, if the cameras are not connected, the user is unable to use any recording options until establishing the connections and pressing “Refresh Camera Connection.” The “Collect Data” tab as well as the side toolbar provides the user with a live preview from the cameras, to initially position the cameras and perform the fine tuning of the focus.

Camera parameters, such as frame size, frame rate, and exposure length need to be set by the software provided by the camera manufacturer and has to be saved in an external configuration file that adheres to the GenICam programming standard. This standard is a generic programming interface that is supported by all compliant cameras and guarantees that a configuration file is transferable between cameras produced by different manufacturers. To set the camera parameters within SnakeStrike, the configuration file must be loaded using the “Load Camera Config” button. Further information, i.e. whether the camera is USB 3.0 or Ethernet-connected, are automatically abstracted. After the configuration of the cameras, the calibration can be started by pressing the “Calibrate Cameras” button that opens the calibration dialogue (Figure 1B). Using the technique described by Li et al. (2013), the intrinsic calibrations, i.e. finding the optical center, focal length, and lens distortion of the individual camera, as well as the extrinsic calibrations, i.e. how the cameras are positioned relative to each other in 3D space, are performed.

### Undistortion and Calibration

The size of the image used for calibration depends on the angles between the cameras and the viewing space covered by the cameras. A large viewing angle and/or a large viewing space requires a calibration image that is sufficiently large to be viewed by more than one camera at a time. Optimal calibration objects allow finding correspondences at multiple levels of resolution. The calibration object must be affixed to a movable planar surface such that the image remains flat, but still can be moved through the cameras’ field of view (Figure 1C). It is not necessary for the calibration image to be fully viewable by each camera or that all cameras see the calibration image at the same time. More important for a successful extrinsic calibration is that the cameras can be linked across images that are shared. For example, in a setup with three cameras (A, B, C), where A and B can see the calibration image in a few image captures, and B and C can see the calibration image in a few captures, it is unnecessary for A and C to also share image captures when capturing the calibration image.

Calibration images should be recorded at low frame rates (e.g. 4 Hz in the current study). This reduces the amount of multiple copies of identical images that would be recorded if the movement of the calibration object is too slow with respect to the camera frame rate. Identical images lead to instabilities in the calibration calculation and unnecessarily increase computation time. Generally, it is recommended to use a slow capture frame rate, and make sure that the calibration object is presented with multiple different orientations relative to the cameras. For a detailed and mathematical explanation of the calibration procedure (see Hartley and Sturm, 1997; Kanatani et al., 2016).

To undistort the images from individual cameras, and to calculate the relative camera positions, we used the technique described by Li et al. (2013). One camera sensor will always be used to define the origin of world space. After collection of the images, the user can choose to see the text output of the calibration and can set the lower boundary for the number of matches. The SURF-like (Bay et al., 2006) difference of Gaussian filter (Li et al., 2013) detectors must find a pair of images that can be paired for further calibration. If the calibration returns with no errors, then a root-mean square error (RMS) of camera positions in space relative to each other is provided (Figure 1B). As the positioning is an optimization and not a closed-formula solution, the error depends on different parameters, such as camera resolution, quality of focusing, number of recorded images, and number of Gaussian filter matches. After calibration, a mask of the experimental setup can be saved (although not required) along with the project information, to improve post-processing such as thresholding.

### Marker Detection and Tracking

After configuration and calibration of the cameras, the “Record” button in the “Collect Data” tab becomes available and the recording can be started. Once a recording has been completed, offline data processing can be started by pressing the “Process” tab. Pressing “Triangulate Points” brings up the dialog window for thresholding the markers from each camera’s image and triangulating those points into world coordinates (Figure 1D). Our marker detection method is analogous to how commercial tracking systems work in that a specific color range, as supplied by the user, is thresholded to detect the markers in an image. This thresholding combined with the grouping of pixels close to each other and then returning the center of that group is how a colored marker on the object/animal is transformed into a marker position. Typically, commercial systems rely on the markers being IR reflective and of a spherical shape to allow the use of ellipse fitting algorithms. Our approach does not have these restrictions. To decrease computational complexity in our software, the initial correspondence of marker position in relation to the different cameras and the colored marker in 3D space are provided by the user. All subsequent correspondences are performed automatically as described below. For thresholding, a range of colors according to the HSV scale can be chosen. To help remove noise, which can pose a severe problem when using this rudimentary approach, a small configurable filter is available. The preview dialog allows the user to fine tune the values for a particular capture session before proceeding to the triangulation. Images being used for triangulation will automatically be undistorted according to the camera distortion coefficients that were calculated separately for each particular camera during calibration.

Markers are not required to be 3D in shape when capturing data, i.e. the IR reflective spheres used by commercial systems, however, when capturing data, it must be ensured that at least two cameras see the tracked points. While two cameras are the minimal requirement for 3D triangulation, the quality of triangulation is considerably improved using three cameras (Stewenius et al., 2005). The benefit of using non-3D markers, like tape or paint, on animals that are difficult to handle such as snakes, is that such 2D markers are more likely to remain attached to the animal. This benefit is offset by the need for more cameras if markers are obscured from one or more cameras. The optimal marker size depends on the camera resolution and size of the object to be tracked. Markers and lighting need to be adjusted to each other. Markers must appear bright enough to allow a dissociation from other low light background colors. On the other hand, markers must not be too bright, because of a potential confoundment with reflections or glare from other surfaces in the experimental environment. We thus recommend choosing colors that have a very high value and saturation in the HSV color space. This facilitates segmenting with both small and large amounts of light. Using a less vibrant color for a marker is possible, but requires that the color is non-glossy; otherwise, reflections on the marker surface can optically change the shape of the marker and thus impair the function of the thresholding algorithm. This could lead to larger errors such as the mid-point of the marker shifting, or even optically splitting the marker into two. In this case, noise smoothing operators in the thresholding dialog are required to rejoin the marker. Accordingly, the larger the markers, the larger the potential error that can occur during triangulation, due to the number of pixels that the marker covers and the problem of finding a representative pixel for this group. When several pixels enter or leave the group, the representative center pixel will most likely change as well. This splitting can also occur because of changes in illumination of the marker, i.e. slight shadow on the marker. This is a source of error related to the lighting of the setup that cannot be fixed by non-flickering lights.

In a perfect situation, where the cameras in a setup have perfect intrinsic and extrinsic calibration matrices, i.e. the parameters of the camera and its position to every other camera is perfectly known, the triangulation of corresponding pixels across cameras is straightforward. A ray extends from each camera sensor through the corresponding pixel in question. In a perfect setup, these projection rays would intersect in 3D space. In reality, however, there are many sources of noise that prevent an intersection of these lines. These sources include noise in the calibration of the camera, noise in how the camera sensor converts light information, noise caused by the viewing of a 3D object from different positions that might not view the object in the same way, etc. When the lines don’t intersect, as is usually the case, a method for finding the 3D point of intersection is required. This means that a new pixel in each image needs to be found such that all the projection lines through those pixels intersect in 3D space. There are many metrics that can be used for determining where this new pixel in each image is located.

To help correct for noise inherent in marker location triangulation we used a technique described by Kanatani et al. (2008) as “optimal correction,” but using the specific implementation from Kanatani et al. (2016), that translocates the marker’s center pixel in the image space a minimal amount such that all projection lines from the cameras intersect again in 3D space. This is known as minimizing the reprojection error (geometric error), i.e. the error in pixel space of the data point and its reprojection. In other words, this method finds a pixel as close to the original pixel in the pixel space for each camera such that the projection lines through those pixels will intersect. Once the lines intersect again, the algorithm of Direct Linear Transformation (DLT) (Sutherland, 1974), which solves using the singular value decomposition (SVD), can be used to calculate the 3D point represented by the corresponding marker locations in each image. If DLT is used without the geometric correction afforded by “optimal correction,” or by another correction algorithm, then the SVD in the equation has multiple possible answers. The algorithm chooses the solution that minimizes the sum of least squares distance, not in the pixel space, but in 3D space from the projection lines of the data points to the point in 3D space that satisfies the intersection constraint. Minimizing the error in the pixel space is generally agreed to be an inferior solution compared to minimizing the error in the geometric space, i.e. the reprojection error (Hartley and Zisserman, 2003) (See Supplementary Videos S1, S2).

When using three or fewer cameras, the globally optimal translocation of data points according to geometric correction can be provided by the polynomial algorithms of Hartley and Sturm (1997) for two cameras, which was later extended by Stewenius et al. (2005) to three cameras. However, as the number of cameras grows, the size of the polynomial function to solve becomes unwieldy. As described by Hartley and Kahl (2007), Stewenius and Nister, in an unpublished work, calculated the degree of the polynomial that would need to be solved for views 2–7. They found 6, 47, 148, 336, 638, and 1081 to be the respective order of the polynomial for calculating the global optimal solution. This shows how quickly the number of local optima of the cost function increases. For more than three cameras, the search for a global optimal solution is commonly done with optimization of a cost function or gradient descent algorithms. These solutions for more than three cameras, however, as mentioned by Hartley and Zisserman (2003) can be quite computationally expensive as well as difficult to program. Quite often these methods also rely on assumptions regarding the source of the noise, e.g. Gaussian distributed. We used the iterative method of Kanatani et al. (2008) to minimize the geometric error without minimizing a cost function or relying on any assumptions regarding the source of the noise. Furthermore, as was shown in Kanatani et al. (2008), since this method starts as an approximation of the solution, it typically requires only a couple of iterations to converge and, in the case of three or less cameras, to provide the same solution as the closed form polynomial equations in less time. In our experience with five cameras the algorithm performs quickly and produces high quality results even with the additional noise that is contributed by the movement of marker center positions. These errors are discussed further in Study 1 below.

**FIGURE 2:**
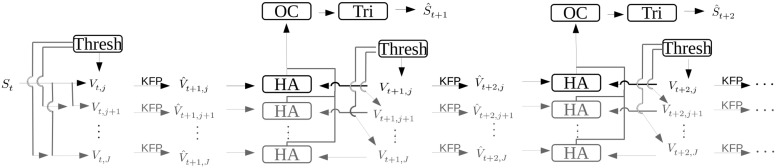
Diagram depicting the flow of data during the processing of images which results in a 3D triangulated point of the markers being tracked. The variable *S* is the set of markers as they exist in the real world with *S*_*t=0*_ being the first time step in the image capture series. The information for initial point correspondence is entered by the user, *via* the labeling GUI. The variable *V*_*t,j*_ is the image pixel position of the markers for time step *t* and camera *j*. Variables with a hat such as $\hat{V_{t,j}}$ or $\hat{S_{t}}$ represent calculated guesses as to the true values *V*_*t,j*_ and *S*_*t*_, respectively. The arrow with the marking “KFP” is a Kalman filter prediction, and the boxes “Thresh,” “HA,” “OC,” and “Tri” indicate the color thresholding algorithm, the Hungarian algorithm, the optimal correction algorithm, and the triangulation algorithm, respectively.

Matching multiple points across multiple cameras is not a trivial problem, as Munkres (1957) demonstrated. If we have M cameras, finding the corresponding point from one camera in the set of all other points in each of the other M-1 cameras is a computationally intensive task. This problem can be solved for two sets in O(N^3^), i.e. the time required to solve the problem scales cubically with the number of inputs, assuming that there are no further constraints to the matching. For more than two sets and when there are additional constraints, such as that the assignment over time stays consistent, these problems are still an active area of research. A general solution to the problem of triangulation of occluded points during tracking has also proved elusive. Only basic methods for the tracking of points are available in our implementation at the moment. Currently, for a triangulation attempt, the user has to select the visible markers in images from a single time step manually using an intuitive GUI. The selection does not need to be pixel precise as the closest visible marker will automatically be selected. This initial marking helps to decrease the computational complexity of assigning matching points across camera views to a more manageable problem. These starting positions are then used as the initial starting positions for points in a Kalman filter (Kalman, 1960). Using this initial configuration, the Hungarian algorithm, also known as the Munkres-Kuhn algorithm (Munkres, 1957; Kuhn, 1959; Bourgeois and Lassalle, 1971), along with the Kalman filter, which receives its step update information from the matching of the Hungarian algorithm, are used to keep the point assignments consistent through time. These algorithms are only used to support the correct assignment of correspondence, while the pixel positions of the marker come from the thresholding and not from the steps of the filter. A high-level diagram of this processing pipeline is shown in Figure 2. The user has the option to force the algorithm to skip timesteps where not all markers for each camera are visible. When the setup consists of more than two cameras, the user can fall back to a set of fewer cameras that have no occlusion for that timestep. An algorithm library for correspondence is used to match corresponding points from images taken at different angles. In the default case this library is the Kalman filter and Hungarian algorithm combination mentioned above. However, since this library is dynamically loaded, the user has the option to write a correspondence plugin for keeping the identity of thresholded points unique through time, making it unnecessary to rely on the basic method described above.

Our framework provides an API where these new algorithms can be supplied to the framework without re-compiling the codebase. This interface is basic in the sense that it provides the points for each timestep, and expects the points to return in ordered lists for each timestep. This creates an interface that puts a minimal amount of constraints on the algorithms that are used to process the point tracking data. More information regarding the API for these algorithms can be found on “https://gwjensen.github.io/SnakeStrike/”. This is where software such as DeepLabCut (Mathis et al., 2018) or LEAP (Pereira et al., 2019) can be integrated into SnakeStrike. This also allows data to be processed by many different algorithms, if necessary. It further offers the possibility for the user to maximize the constraints such as how to handle obscured points, what to do when point labels are swapped, etc.

### Viewing Tracking Data

**FIGURE 3:**
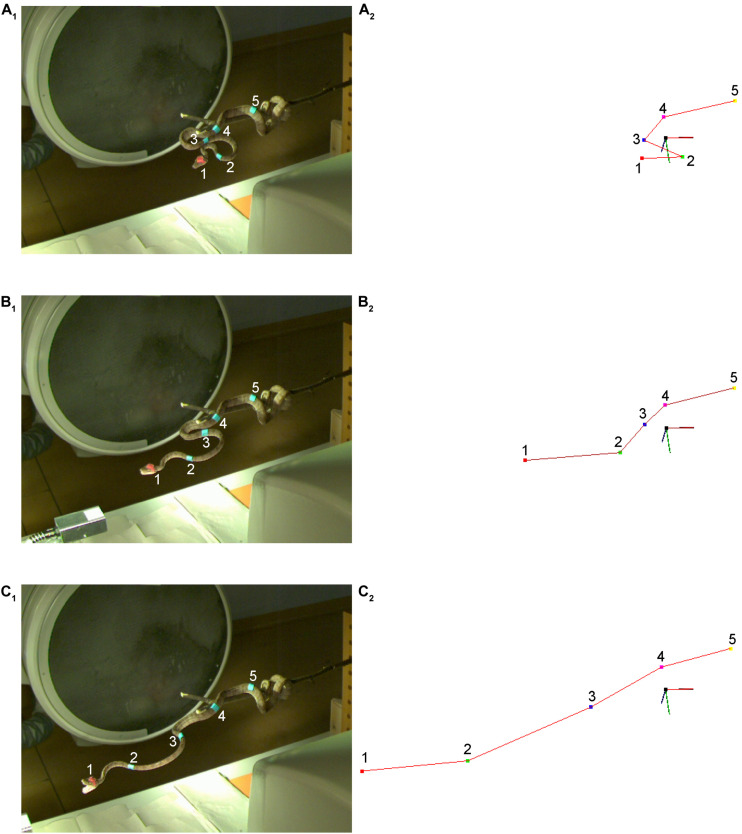
Experimental setting for capturing and analyzing snake strikes in study 2. **(A–C)** Raw images of a strike of an amazon tree boa (*Corallus hortulanus*) at three different time steps: at a stationary position **(A_1_)**, and approximately in the middle of the strike **(B_1_,C_1_)**. The large black circle behind the snake is the horizontal *X*-ray emitter, while the vertical *X*-ray emitter above the snake is out of view. Corresponding triangulation is plotted as output by the SnakeStrike software for the corresponding images **(A_2_–C_2_)**. The coordinate system has been rotated such that triangulated points and images have the same viewpoint. Each marker (1–5) on the snake is depicted by a different color label in the triangulated data, starting with the red (1) point and ending with the yellow point (5) in **(A_2_–C_2_)**; a red line connects all points in the triangulation. This line corresponds to the marker positions on the snake from rostral to caudal and is not based on pose estimation. The direction of the line is defined by the user during triangulation. The data shown in plots **(B,C)** are 86 images (114.7 ms) apart.

After triangulation, the data can be viewed by the built-in 3D point cloud viewer. This generates an animation of the movement through time, and also allows stepping through each individual timestep. A line connects the points in the order as indicated by the user for disambiguation. This line is useful for relating triangulated points to the body of the animal, but does not directly reflect pose information of the animal. For example, in the case of a snake, the line won’t follow the contours of the snake unless the markers are spaced with minimal distance to each other. The points are also of different colors to prevent ambiguity when viewing the motion of the points through time (Figures 3A_2_,B_2_,C_2_). The triangulation performed by SnakeStrike does not perform any direct filtering or smoothing on the triangulated points over time. It only provides a simple forward-backward filter as part of the GUI window to allow the user to see how filtering or smoothing could improve the data.

### Procedures for Study 1: Measurement of Accuracy

The accuracy of SnakeStrike was determined under particular control conditions, that, however, differed with respect to the experimental setting used in study 2 for the strike movements of snakes (e.g. cameras, lighting conditions, etc.). The accuracy of the system was determined with a robotic arm that reproducibly moved an artificial object to calculate the error level for the calibration technique and illustrated the effect of different recording conditions. The setup consisted of a robotic arm (Model: “Franka Emika Panda,” Franka Emika GmbH, Munich, Germany) used to move a flat plane with affixed markers in a specific spatial configuration (Figures 1E,F and Table 1). Robotic arm motion allowed for precise movements with identical trajectories, while parameters such as speed were altered under defined conditions. With this approach two types of camera lenses, two different marker dimensions, and two different movement speeds were tested. The definition of the exact distances between the points allowed calculating the error in 3D triangulation between all points. The affixation of the points to a plane allowed calculating the error of the points from the plane that fits all points with the least error.

**TABLE 1:** Distance between markers on the calibration object used for Study 1.

	1	2	3	4	5
**Small mark number**					
1	0	72.0	80.5	101.8	72.0
2		0	36.0	72.0	101.8
3			0	36.0	80.5
4				0	72.0
5					0
**Big mark number**					
1	0	84.0	93.9	118.8	84.0
2		0	42.0	84.0	118.8
3			0	42.0	93.9
4				0	84.0
5					0

*Measurements are all in mm.*

The basic experimental setting was as follows: placement of three cameras, oriented around the robotic arm (Figure 1F). Bright neon green markers (small: 10 mm × 10 mm; large: 22 mm × 22 mm) were attached at specific locations on a flat piece of medium-density fiberboard to ensure that the markers were aligned in the same spatial plane; the chosen marker color was unique and did not occur on objects anywhere else in the cameras’ field of view. For the Fujinon DV3.4x3.8SA-1 (Fujifilm, Tokyo, Japan) lens, the distance between cameras and marker plane was about 110 cm (Figure 1E). For the Ricoh FL-CC0614A-2M (Ricoh, Tokyo, Japan) lens, the distance was increased to ∼175 cm, because of the longer focal length of the lens. As the cameras had to be moved, calibration differences as well as lens differences were tested. Each capture session was completed with the cameras set to an image size of 640 × 480 pixels at a frame rate of 750 Hz. For each capture, 6000 images were recorded. For triangulation, the thresholding viewer tools of SnakeStrike were used and set to a color range with the most correct detection of the markers and minimum false-positive detections.

### Procedures for Study 2: Strike Movement of the Amazon Tree Boa (*Corallus hortulanus*)

The tracking framework was applied for the first time ever on living animals in combination with biplanar *X*-ray to capture fast snake strikes. The field of view of the *X*-ray tubes was too small to encompass the entire strike of the snakes while still providing adequate resolution. Therefore, an additional data capturing method was required to compare local information obtained from the biplanar *X*-ray motion capture with global snake movement information. This multi-modal data and the analysis of the resulting data fusion is not a component of the tracking system, and thus out of scope. Nevertheless, we were able to assess the usage of the system with live animals and to demonstrate that this stand-alone 3D motion tracking system can easily be integrated in existing experimental set-ups to record multi-modal data sets.

The experiment included four amazon tree boas (*Corallus hortulanus*) with a snout-vent-length (SVL) of 100–120 cm. Snakes of either sex and a body mass of 23–69 g were obtained from the in-house animal breeding facility at the Chair of Zoology at the Technical University of Munich. Snakes were kept at a temperature of 22–30°C on a 12 h:12 h light:dark cycle. Permission for the experiments was granted by the respective governmental institution for animal welfare (Thüringer Landesamt für Verbraucherschutz; code: 15-003/16). For the experiments, five cameras running at 750 Hz at a spatial resolution of 640 × 480 pixels were used resulting in 7500 images per camera per capture sequence. This required ∼11GB RAM (We used the Bayer BG8 image format) and ∼7 GB disk space to record and store one capture, excluding any memory or storage space to run SnakeStrike. The orientation of the snake in space when anchored to a branch represented a difficult condition to reliably capture images as snakes can coil back onto themselves, thus potentially occluding markers. Additionally, the placement of markers on the snake, though spaced out along the body of the snake, can end up next to each other when the snake forms its characteristic S-shaped curves. Therefore, multiple cameras were necessary such that at least two cameras saw the markers at any timepoint.

Avery No. 3320 multipurpose labels (Avery Dennison Corporation, Glendale, CA, United States) were used as markers as they can be stained with any suitable color, and have a good adhesion, without irritating the skin. The labels were painted in a light blue color as it would be the only incidence of that color in the experimental setting. There were blue markers on the body and an additional red marker on the head because the head becomes obscured by the opening of the mouth as described by Cundall and Deufel (1999) and the *X*-ray tubes did not allow for setting up cameras directly above the snake (Figures 3A_1_,B_1_,C_1_). To reduce errors and to avoid correspondence switching of the markers, the body and head markers had different colors. Accordingly, the two marker colors were triangulated separately and then required post-processing to fuse the data manually using a simple script.

## Results

### Study 1: Measurement of Accuracy

**FIGURE 4:**
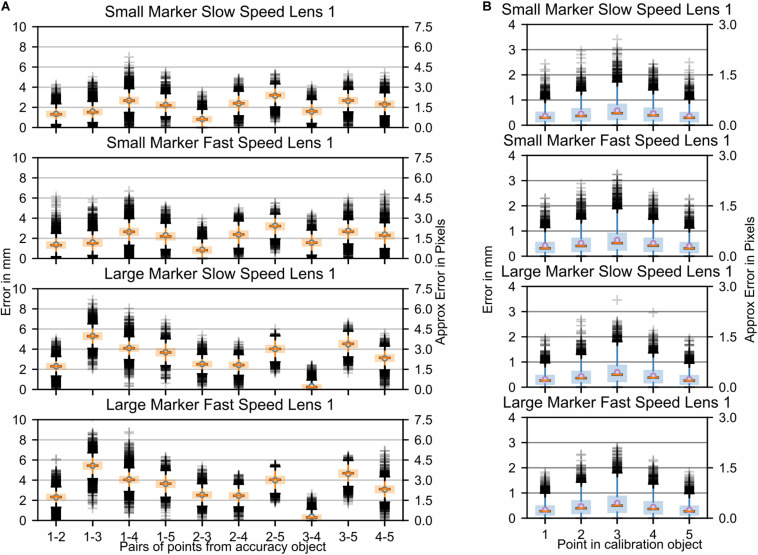
Study 1: Error in marker location while the fiberboard with attached markers was moved through space by the Franka Emika Panda robotic arm. Parameters: two marker sizes and two relative speeds of movement of the markers. Data is shown for one camera lens (Fujinon DV3.4x3.8SA-1) only. In Supplementary Figure S1 additional data recorded with a second camera lens (Ricoh FL-CC0614A-2M) are presented. **(A)** Each boxplot refers to an interpoint distance error between two points of known distance in the calibration object (Table 1); the numbers on the *x*-axis represent the points and are separated by a hyphen. **(B)** Marker distance from the best-fitting plane for all markers.

The accuracy of the tracking system was determined by using a robotic arm for the generation of a movement of the flat plane through the visual field of the cameras (Figures 1E,F). Based on the variations of the experimental protocol, it was possible to determine the errors related to the different configurations. To provide an intuitive understanding of the performance abilities of the system, we also converted errors from absolute 3D world space measurements to approximate pixel space equivalencies. Figure 4A (Supplementary Figure S1A) and Figure 4B (Supplementary Figure S1B) illustrate the errors resulting from running the same movement at two different speeds, two different lenses, and two different marker sizes. The faster robotic arm speed covered the same motion trajectory as the slower speed, but also covered a slightly different motion at the end due to the faster movement. This was expected as it produces the same trajectory as the movement at the slower speed, but over a shorter amount of time. Details of the results with different marker size, lenses, and movement speed were plotted in the various rows and columns in Figure 4 and Supplementary Figure S1. A major outcome of these experiments was the observation that neither the change of lenses nor the speed of the movement has a substantial effect on the accuracy of the reconstruction. The latter finding was also not too surprising given that the movement speed was far slower than the camera frame rate (750 Hz) for these experiments.

**FIGURE 5:**
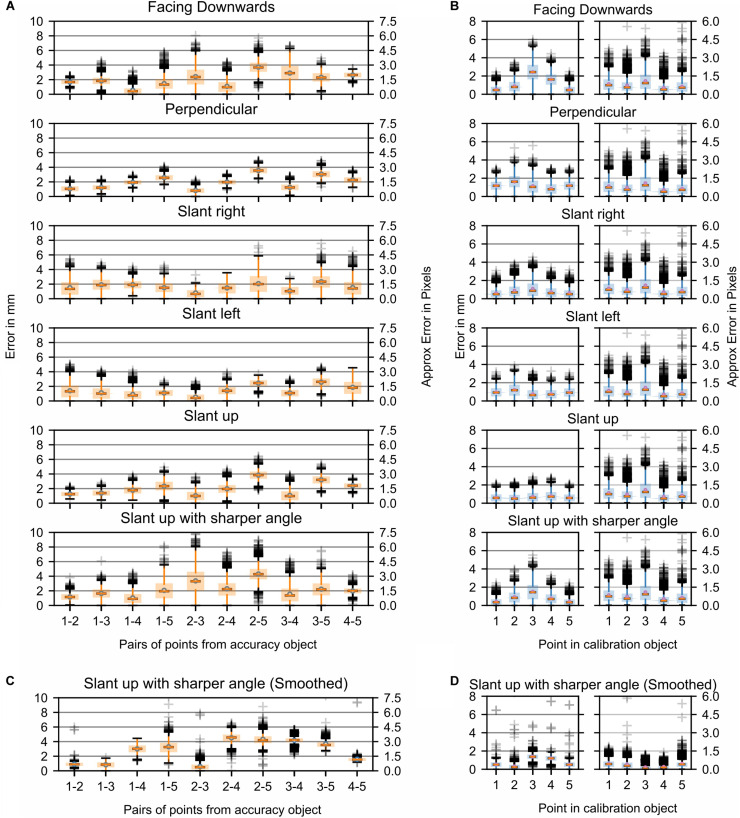
Study 1: Error in marker location with a stationary fiberboard across 6000 time steps. **(A)** Stationary markers viewed from different angles by the center camera. Each boxplot refers to an interpoint distance error between two points of known distance in the calibration object (Table 1). The numbers on the *x*-axis represent the points and are separated by a hyphen. **(B)** Distance of markers from the best-fitting plane for all markers (left). Movement of triangulated points in 3D from their mean while markers were stationary (right). **(C,D)** Same data as shown in **(A,B)** but post-processed with a Kalman filter. For plots of the remaining data post-processed with a Kalman filter (see Supplementary Figure S2).

The largest visibly observed differences were found for the size of the markers, with an increase of ∼2–3 mm (∼2 pixels) in the average error per point when using larger markers. This was likely due to the fact that the larger the marker, the more the center of the marker potentially shifts. This shifting can be caused by parameters such as changes in illumination, color intensity, or visibility. It is noteworthy, however, that the planar error did not change between the two marker sizes. In order to better understand the effect that aspects such as viewing angle, lighting, or color intensity changes have on the accuracy, stationary images were collected. Figures 5A,B show the respective distance errors between points and the distance from the closest fitting plane, respectively. During all captures, 6000 images were acquired at a frame rate of 750 Hz. In this case, small markers were used and each plot represented the position of the fiberboard relative to the main camera. The positions were as follows: “facing downwards,” “facing perpendicular,” “slanted right,” “slanted left,” “slanted up,” and “slanted up with a sharper angle.” It is noticeable that the error ranges for “slant right,” “facing downwards,” and for “slanted up with a sharper angle” were considerably larger than for the other capture angles. Variances for both of these positions ranged mostly from 4 to 6 mm (∼3–4.5 pixels), while other positions tended to have less than 4 mm (∼3 pixels) of variance. The markers perpendicular to the camera as well as the “slant left” capture error ranges were considerably smaller than reported for moving markers. Markers at other angles relative to the camera had error variances comparable to those during motion captures.

To better explain the source of these errors in the triangulation, the original pictures were reanalyzed to potentially discern differences between the positions that showed a smaller error variance and those that showed a larger variance. The first noticeable difference was that the angle of the marker board relative to the camera was larger in the “slanted right” compared to the “slanted left” capture configuration. A similarly sharp angle was also present relative to one of the cameras in the “slanted up with a sharper angle” capture condition. Thus, the sharper the angle of the camera optical axis relative to the marker, the smaller the marker from the view of the camera. The resultant smaller viewable marker size combined with color intensity differences due to the angle relative to the camera caused the center point of the marker to move with either the flickering of the light source or the change in illumination caused by the angle. Part of the error was likely due to the fact that the triangulated points provided by SnakeStrike have not been filtered or smoothed through time. To demonstrate the effect that a filter would have on the error variance, the data from Figure 5 were processed by a simple Kalman filter (Kalman, 1960). As illustrated in Figures 5C,D and Supplementary Figure S2, it was clear that the use of such a filter drastically reduces the error variance for both the interpoint error as well as the planar error, indicating the necessity to apply such a simple and easy to implement post-processing to obtain even more reliable motion tracking.

Analysis of the data collected from this experimental paradigm showed that even though a high frequency fluorescent bulb was used as the main light source, a minor oscillatory flicker occurred in the image sequence. This introduced a noticeable effect on the accuracy of marker positions as the flicker significantly changed the color characteristics of the markers with respect to saturation and hue. To precisely quantify the error, introduced by the flickering light, a second test of the accuracy object was performed in a new setup where the object remained stationary in a position that was perpendicular to the center of three cameras. In this second experimental setting, data was recorded using a completely separate location with a bright light source consisting of four AQ Aquaflora 54-watt fluorescent bulbs that did not produce any light flickering, and the spacing of the cameras was similar to the original setting. The respective data are presented in Supplementary Figures S3B,C, S4B,C and give a clear approximation of the error that the flickering of the light has introduced in the originally collected data (Supplementary Figures S3A, S4A), i.e. an average spatial error difference of maximally 1 mm (∼1 pixel) with considerably increased variances, 2–3 mm (∼2 pixels) for the flickering data error. As the errors for both the flickering and the non-flickering light condition show the same trends, there is still a systematic error in the triangulation of the system. Given that the data obtained in the flickering and non-flickering light condition used different camera calibrations, it is more likely that the residual error derived from inaccuracies in the calibration object, or an error in the triangulation and rudimentary thresholding algorithms, though it is nevertheless still small in magnitude.

### Study 2: Strikes of the Amazon Tree Boa (*Corallus hortulanus*)

**FIGURE 6:**
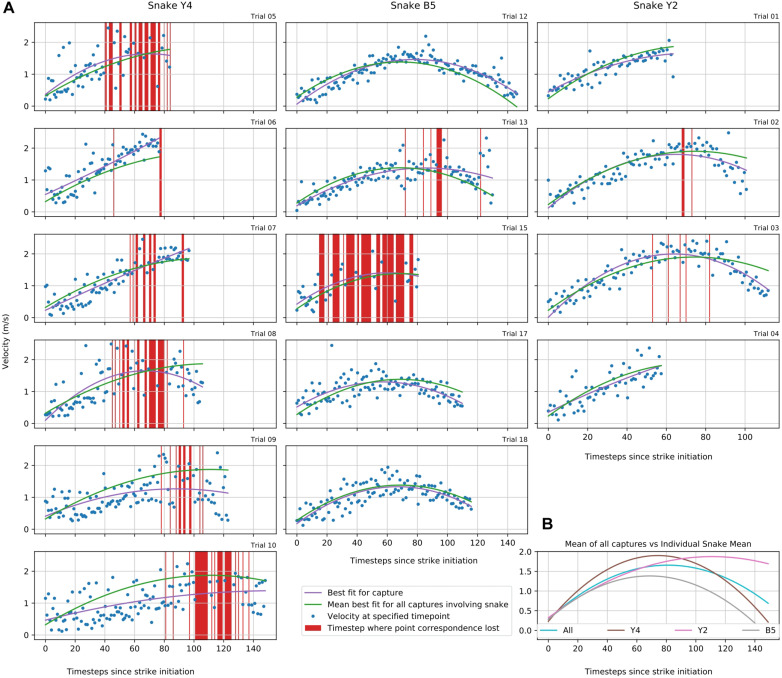
Study 2: Snake strike velocity over time **(A)** 3D positions in study 2 of each snake’s head for each trial are plotted to illustrate the velocity changes during the strike. In some trials, e.g. Y4 markers were obscured from the view of at least one camera resulting in a loss of correspondence as a new and usually temporary point was assigned to be the marker location. These time steps were removed using a velocity threshold of 2.5 ms^– 1^ (red vertical bars). **(B)** Group aggregate velocity statistics for all snakes individually and as mean across all snakes tested in this study.

In the framework of the experimental setting, 20 snake strikes were recorded, out of which 15 were used to provide tracking data that could be fully processed by SnakeStrike (Figure 6). The meta-information regarding successful strikes is presented in Table 2. Captures 19 and 20 derived from a smaller snake and thus were not included. Captures 14 and 16 used a rather strong heat element as infrared target to elicit the strike instead of the IR emitter that was used for the other strikes. This strong heat element produced visible light that affected the ability of SnakeStrike to properly track the markers. Capture 11 did not yield a consistent set of images, likely because of partially missing data from one of the 5 cameras and therefore was excluded from further analysis. Details regarding the velocity of each strike over time is presented in Figure 6. The dots indicate the calculation of the velocity for each timestep using 3D triangulation information. The value for each individual dot was calculated as the change in distance between two subsequent timesteps, where a timestep was denoted by a captured image. There are two lines for each capture plot with one showing the best fit for the trial data and the other indicating the average best fit for all trials of a particular snake. Data points that did not allow an analysis were removed from the plot (red bars) based on a velocity threshold of 2.5 ms^–1^. This omission of data typically occurred when the identity of points was swapped for a few timesteps, or when the thresholding process found a different reflection or source of color than the marker in question and assigned a new – but often only temporary – point as marker for that timestep. An example for the velocity change of a snake strike is plotted at the lower right of Figure 6 as the best fitting curve for all snake strike trials in comparison to the best fitting curve for all trials of each individual snake.

**TABLE 2:** Strike meta-data as calculated from initial forward movement of strike until start of head retraction.

Trial	Snake	Duration (s)	Distance (m)	Speed (ms^–1^)	Max Velocity (ms^–1^)
1	Y4	0.0866458717	0.0958	1.1055	1.6393
2	Y4	0.1359673678	0.1731	1.2732	1.8036
3	Y4	0.1506305153	0.2136	1.4182	1.9968
4	Y4	0.0786477912	0.0829	1.0542	1.8137
5	Y2	0.1146391533	0.1461	1.2743	1.6408
6	Y2	0.1066410728	0.2192	2.0556	2.3136
7	Y2	0.1306353142	0.1472	1.1270	2.1715
8	Y2	0.1426324349	0.1567	1.0984	1.6513
9	Y2	0.1652936629	0.1419	0.8583	1.2661
10	Y2	0.1986189981	0.1573	0.7920	1.3873
12	B5	0.1959529713	0.1901	0.9700	1.4633
13	B5	0.1746247567	0.1695	0.9705	1.3763
15	B5	0.1093070996	0.0968	0.8854	1.4035
17	B5	0.1479644885	0.1462	0.9878	1.3030
18	B5	0.155962569	0.1468	0.9411	1.3333
All Mean		0.1396109378	0.1522	1.1208	1.6376

## Discussion

As indicated previously for machine vision in social interaction studies (Robie et al., 2017), when individual animals are visually indistinguishable, the task of tracking in the presence of occlusions or partial overlap is an unresolved problem. For snake motion tracking, a similar problem exists as positions on the body surface of the snake are nearly identical, such as when the snake skin consists of a repetitive pattern. This makes markerless tracking methods, such as DeepLabCut (Mathis et al., 2018), much harder to employ successfully on patterned snakes. Markerless annotation programs such as DeepLabCut further require a set of manually labeled images to train the software. In the case of DeepLabCut, this training can require a couple of hundred images per camera, though in practice a smaller number can be sufficient, and if the camera is moved, it might require re-labeling of a set of new images from that camera. Furthermore, DeepLabCut does not provide 3D triangulations of annotated points. On the other hand, non-patterned skin can also be problematic as large sections of the snake surface look very similar and indistinguishable causing specific positions to be difficult to discern with sufficient spatial resolution. With the snake moving and changing body shape in a very short period of time, the labels assigned to these markers will be swapped in most cases, unless the system is able to uniquely identify and track a particular marker over time. This is implemented in many systems by using multiple markers as a single marker and putting them into a unique configuration scheme that is identifiable by the system (Theunissen et al., 2017), thus filling up even more skin space with markers. Unfortunately, the maintenance of the spatial configuration can be also very problematic since some animals (e.g. insects or small birds) are too small to carry an extra payload or are able to remove these markers very easily (e.g. snakes or squids). For example, a snake easily twists itself and effortlessly removes even the smallest markers. This is the major reason why studies such as that by Theunissen et al. (2017) used rigid structures attached to the body and head of the animal as markers. This method allows motion capturing of the whole animal as body and head can be tracked even when there is occlusion of some markers. In a large system with many cameras the triangulation is robust to marker loss through the view of many cameras. Unfortunately, this method is not suitable when attempting to capture the kinematics of the animal or the kinematics of particular appendages.

SnakeStrike is a framework that allows researchers in animal tracking to use one piece of software from experiment start to finish and in combination with high-speed cameras to save time in the processing of 3D triangulation data for animals that can be difficult to track. Our preliminary data set on boid snakes showed that this framework is suitable to track even fast movements such as snake strikes and provides first information about the instantaneous speed during the complete strike of a boid snake. Although this data set primarily serves to demonstrate potential applications of this framework, it already showed that instantaneous strike speed has a similar magnitude, when compared across different individuals. In addition, our data on boid snakes shows similar strike profiles and trajectories as described for strikes of viperid snakes (Kardong and Bels, 1998; Herrel et al., 2011), suggesting the presence of a common motor program for executing strike behavior. Similar experiments with an increased number of markers placed on the snake body, would also allow for a further, more detailed analysis of the contribution of typical loop formations in strike progression.

The data presented in Study 2 shows that the new framework presented here allows for collection of data even from vastly agile animals without the necessity of purchasing costly commercial systems or having to combine multiple other software solutions. Despite room for further improvement, the system provides scientists with new options and another alternative to existing systems such as DLTdv (Hedrick, 2008) to record novel data sets. Some drawbacks of the system derive from the necessity to simultaneously save the images from multiple high-speed cameras, which requires a large amount of memory for the initial capture, high CPU load for processing, and a large amount of hard drive space for long term storage. This requires a large upfront cost for a computer, although the computer can also be used for other tasks, and still costs only a fraction of a commercial system. However, the storage of the raw data, allows recalculations and reanalysis at any time (see below). Since changes of light intensity, shadowing, and occlusion of markers can occur within a given recording session, thresholding color from the images can be complicated and time consuming. The impact of this issue can be reduced, though not completely eliminated, by pre-tests of the color(s) to be thresholded in the actual setting, as well as by strict adherence to a consistent experimental environment and regime.

The benefits of this framework considerably compensate for the few disadvantages, also because in many experiments, the latter can be at least partially circumvented. The reduced upfront costs compared to commercial motion tracking systems allows greater ability to incorporate motion tracking at high speed in animal studies. As shown in Study 1, the error in the system is small with regard to normally occurring sources of errors such as flickering lights, color thresholding problems, or marker identity swapping. For animals in which affixing large 3D markers is impossible because the animal might remove the markers or they do not remain affixed, this is a particular improvement. In studies of animal behavior, the goal usually is to obtain the largest amount of usable data as possible. Being able to store and reassess all originally captured images, rather than having to only rely on calculated 3D points is a very big advantage, since various additional analyses can be performed offline. This indicates that the acquired information can be used not only for the initial, principal aim of a project, but also allows answering novel questions without the necessity to perform a second experiment. Since all 3D points are decoupled from the images, the generation of the points in terms of decrease in error can be improved by new methods in the future. The data generated from older studies can therefore be re-interpreted or interpreted in greater detail, when, for example, new algorithms for coping with occlusion of tracking markers has been developed. Triangulation of data collected today would suffer from this aspect of the current state of the art in algorithms. However, if this problem is improved, these data can be easily re-interpreted with new algorithms and possible new insight can be obtained, without having to re-run tedious and often difficult and time-consuming experiments. SnakeStrike brings the functionality of several open source projects together in a way that is highly beneficial to researchers who have no access to expensive motion capture systems. Researchers who work with non-standard and especially fast-moving animals now have an affordable option to exploit novel experimental ideas. In addition, those interested in testing new algorithms for object correspondence over time can generate real-world data sets very quickly and easily, or test ideas on previously collected data.

## Data Availability Statement

The datasets generated for this study are available on request to the corresponding author.

## Ethics Statement

The animal study was reviewed and approved by the Thüringer Landesamt für Verbraucherschutz; code: 15-003/16.

## Author Contributions

GJ, HS, PS, and TK: conceptualization. GJ: methodology, software, validation, investigation, data curation, writing – original draft. GJ, HS, EH, PS, and TK: formal analysis, writing – review editing. PS and TK: resources. GJ and TK: visualization. HS, EH, PS, and TK: supervision. TK: project administration. HS, PS, and TK: funding acquisition. All authors contributed to the article and approved the submitted version.

## Conflict of Interest

GJ and PS were employed by Volkswagen Group, in the Machine Learning Research Lab. The remaining authors declare that the research was conducted in the absence of any commercial or financial relationships that could be construed as a potential conflict of interest.

## Footnotes

1 http://www.compadre.org/OSP/

## Supplementary Material

FIGURE S1 Study 1: Error in marker location while the fiberboard with attached markers was moved through space by the Franka Emika Panda robotic arm. Parameters: two marker sizes and two relative speeds of movement of the markers. Data is shown for one camera lens (Ricoh FL-CC0614A-2M) **(A)** Each boxplot refers to an interpoint distance error between two points of known distance (Table 1) in the calibration object; the numbers on the *x*-axis represent the points and are separated by a hyphen. **(B)** Marker distance from the best-fitting plane for all markers.

FIGURE S2 Same data as shown in Figures 5A,B, but post-processed with a Kalman filter to illustrate the improvement by filtering or smoothing of the triangulated data for the analysis. **(A)** Each boxplot refers to an interpoint distance error between two points of known distance in the calibration object (Table 1). The numbers on the *x*-axis represent the points and are separated by a hyphen. **(B)** Distance of markers from best-fitting plane for all markers (left). Movement of triangulated points in 3D from their mean while markers were stationary (right).

FIGURE S3 Study 1: Effect of light-flickering on inter-marker distance error variance. The markers were stationary and perpendicular to the cameras’ position. Each boxplot refers to an interpoint distance error between two points of known distance in the calibration object (Table 1). The numbers on the *x*-axis represent the points and are separated by a hyphen. **(A)** Example of a trial where flickering was present in the light source. **(B)** Example of a trial in a different setting where light-flickering was absent. **(C)** Example of a trial in the same setup as in **(B)**, but with a different camera calibration.

FIGURE S4 Study 1: Same data as in Supplementary Figure S3, but with the error displayed as the distance of the marker from the best-fitting plane for all of markers. **(A)** Example of a trial where flickering was present in the light source. **(B)** Example of a trial in a different setup where light-flickering was absent. **(C)** Example of a trial in the same setup as in **(B)**, but with a different camera calibration.
