# Supplementary figures and images for: SnakeStrike: A Low-Cost Open-Source High-Speed Multi-Camera Motion Capture System
Source: Front Behav Neurosci. 2020 Aug 3;14:116. doi: 10.3389/fnbeh.2020.00116 (PMC7416652; doi:10.3389/fnbeh.2020.00116)

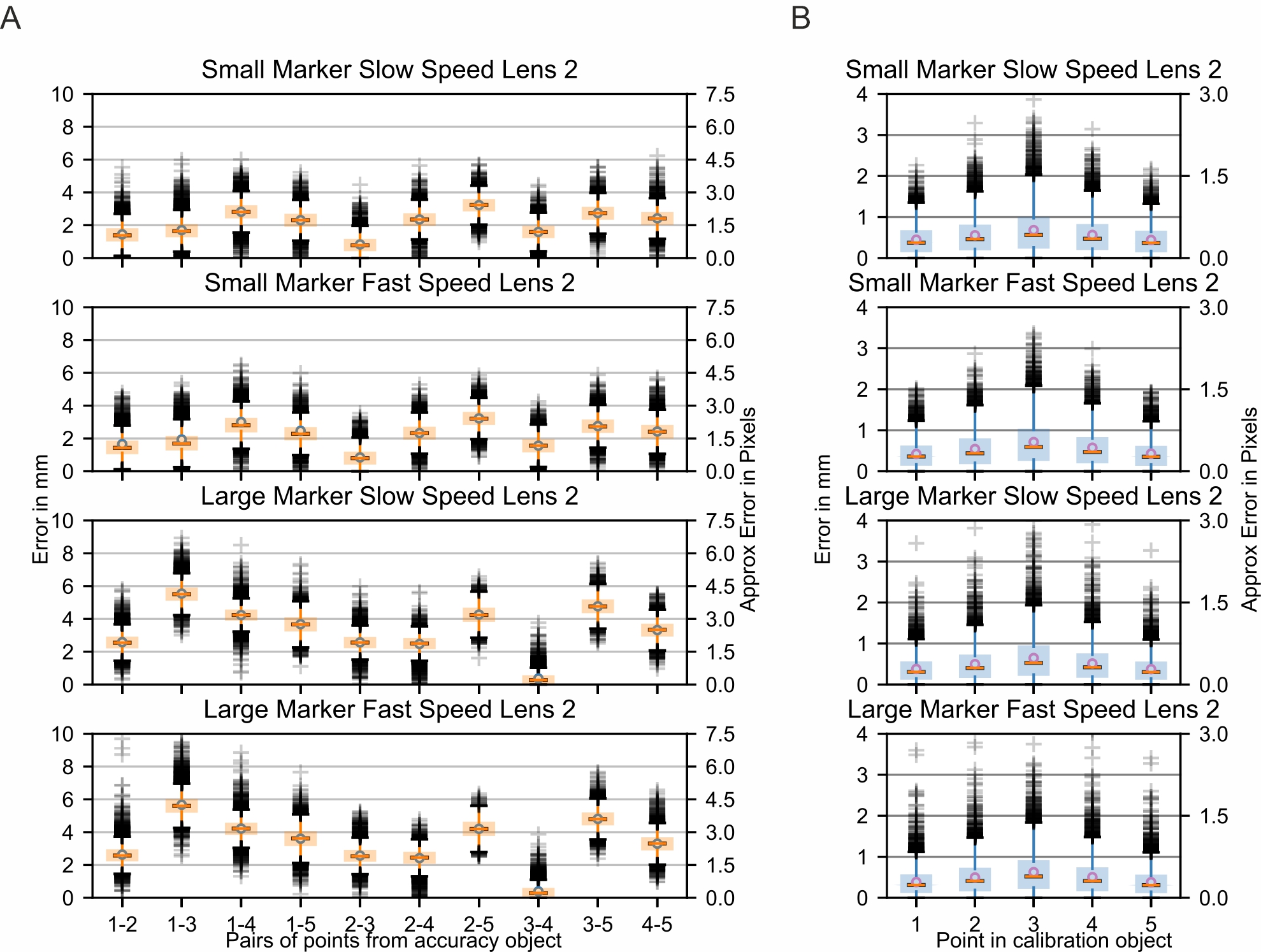

Supplement: FIGURE S1 — Study 1: Error in marker location while the fiberboard with attached markers was moved through space by the Franka Emika Panda robotic arm. Parameters: two marker sizes and two relative speeds of movement of the markers. Data is shown for one camera lens (Ricoh FL-CC0614A-2M) (A) Each boxplot refers to an interpoint distance error between two points of known distance (Table 1) in the calibration object; the numbers on the x-axis represent the points and are separated by a hyphen. (B) Marker distance from the best-fitting plane for all markers. [file Image_1.JPEG]

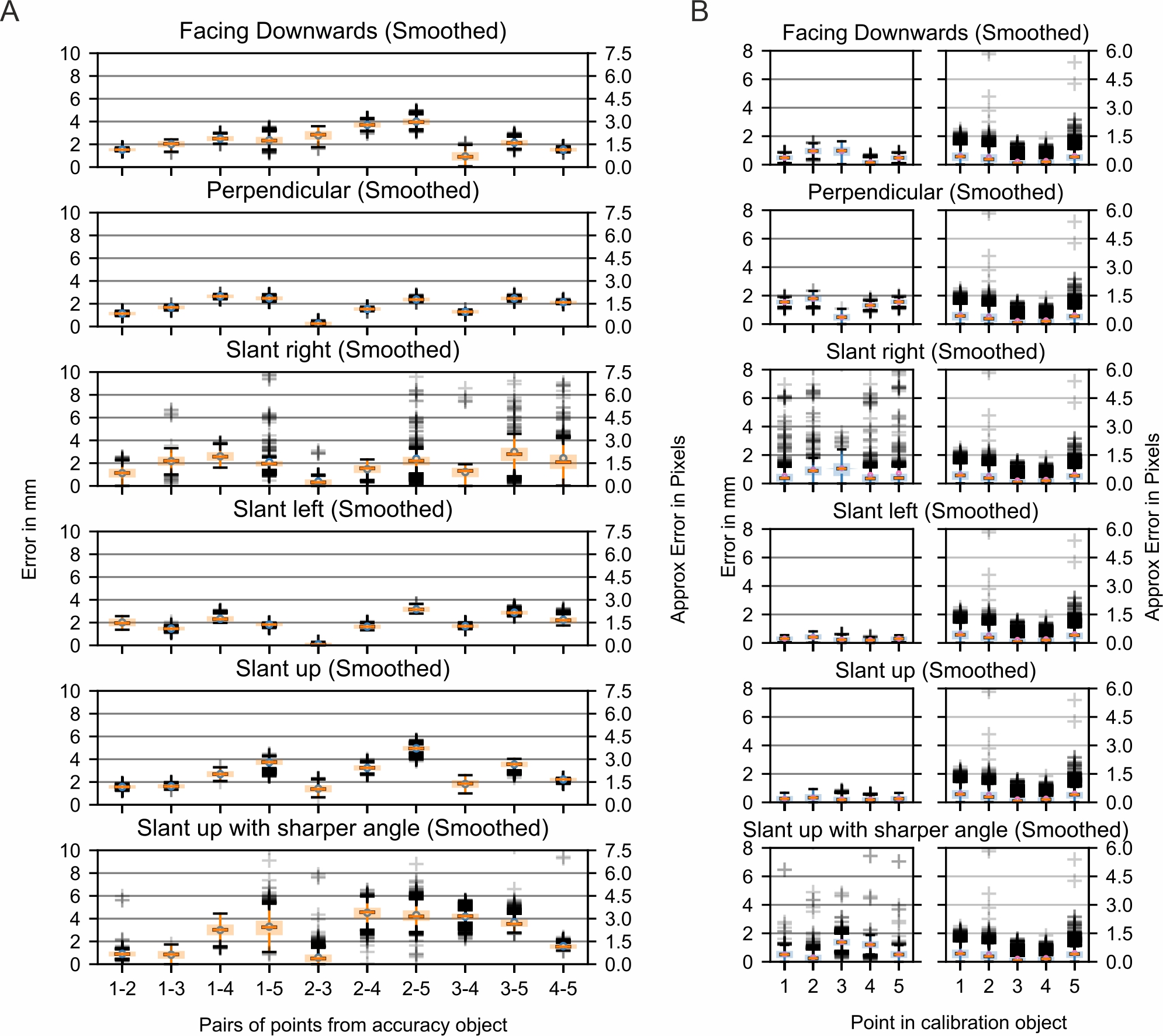

Supplement: FIGURE S2 — Same data as shown in Figures 5A,B, but post-processed with a Kalman filter to illustrate the improvement by filtering or smoothing of the triangulated data for the analysis. (A) Each boxplot refers to an interpoint distance error between two points of known distance in the calibration object (Table 1). The numbers on the x-axis represent the points and are separated by a hyphen. (B) Distance of markers from best-fitting plane for all markers (left). Movement of triangulated points in 3D from their mean while markers were stationary (right). [file Image_2.JPEG]

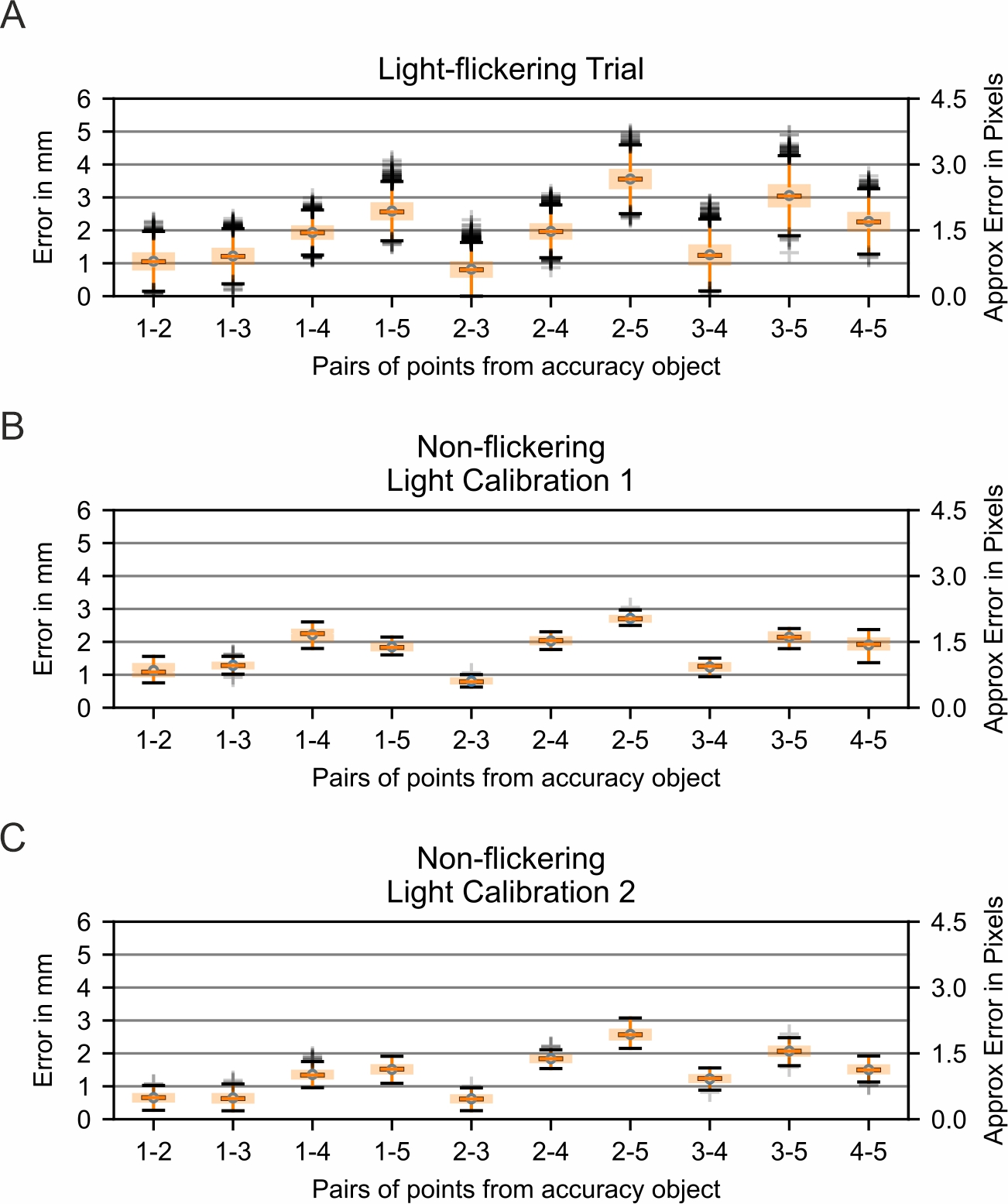

Supplement: FIGURE S3 — Study 1: Effect of light-flickering on inter-marker distance error variance. The markers were stationary and perpendicular to the cameras’ position. Each boxplot refers to an interpoint distance error between two points of known distance in the calibration object (Table 1). The numbers on the x-axis represent the points and are separated by a hyphen. (A) Example of a trial where flickering was present in the light source. (B) Example of a trial in a different setting where light-flickering was absent. (C) Example of a trial in the same setup as in (B), but with a different camera calibration. [file Image_3.JPEG]

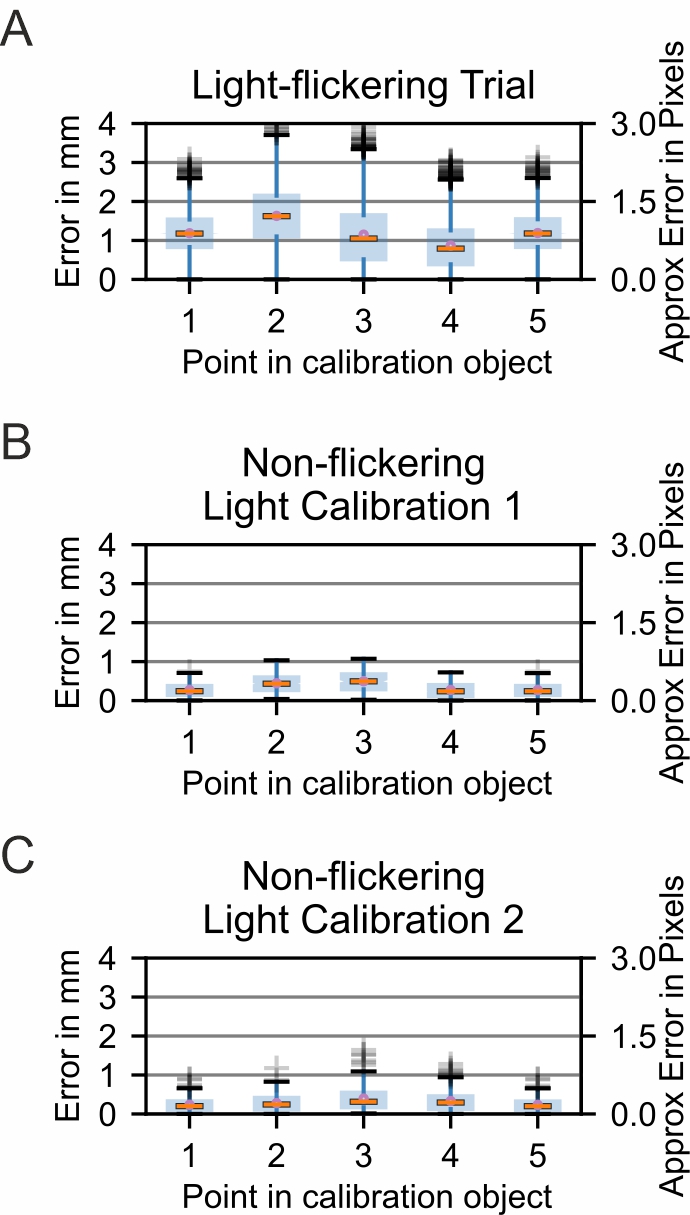

Supplement: FIGURE S4 — Study 1: Same data as in Supplementary Figure S3, but with the error displayed as the distance of the marker from the best-fitting plane for all of markers. (A) Example of a trial where flickering was present in the light source. (B) Example of a trial in a different setup where light-flickering was absent. (C) Example of a trial in the same setup as in (B), but with a different camera calibration. [file Image_4.JPEG]
